# Resilience Mediates the Relationship Between Social Support and Quality of Life in Patients With Primary Glaucoma

**DOI:** 10.3389/fpsyt.2019.00022

**Published:** 2019-01-31

**Authors:** Yiwen Wang, Ying Zhao, Sisi Xie, Xinru Wang, Qing Chen, Xiaobo Xia

**Affiliations:** ^1^Department of Ophthalmology, Xiangya Hospital, Central South University, Changsha, China; ^2^The Eye Institute of Central South University, Changsha, China; ^3^Health Management Center, Xiangya Hospital, Central South University, Changsha, China

**Keywords:** glaucoma, resilience, social support, quality of life, mediator

## Abstract

**Objective:** Glaucoma is a serious disease causing blindness, which seriously affects the quality of life of patients. Previous studies have reported that both resilience and social support play important roles in enhancing the quality of life of patients with some diseases. The purpose of this study is to investigate if resilience mediates the relationship between social support and quality of life in patients with primary glaucoma.

**Methods:** We conducted a cross-sectional study with 120 patients with primary glaucoma in China. The Connor-Davidson Resilience Scale was used to measure resilience, and social support was measured by the Perceived Social Support Scale. The Glaucoma Quality of Life-15 questionnaire was used to measure quality of life.

**Results:** There were significant positive correlations between all dimensions and total scores on the resilience, social support, and quality of life scales (*p* < 0.01). Both resilience and social support could predict these patients' quality of life. A bootstrap test showed that resilience had a partial mediating effect on the relationship between social support and quality of life (*p* < 0.05).

**Conclusion:** Resilience mediates the relationship between social support and quality of life in primary glaucoma patients. This finding suggests that increasing resilience and social support can improve the quality of life of primary glaucoma patients in clinical practice.

## Introduction

Glaucoma is a common blinding ophthalmic disease featuring pathologically elevated intraocular pressure that leads to visual dysfunction and ocular tissue damage (1, 2). Considering this chronic disease has a potential blinding outcome, it can often cause a psychological burden to patients (such as stress, anxiety, fear, and depression) (3–5). These emotional responses can significantly impair quality of life outcomes (3, 5–7). The quality of life of glaucoma patients is an important indicator for evaluating the efficacy of glaucoma treatment (8–11).

Resilience is a good adaptability of individuals in the face of adversity in life, trauma, tragedy, threat, or other major pressures (12, 13), and resilient individuals “bounce back” from stressful experiences quickly and effectively (14). Studies have shown that there is a positive correlation between quality of life and resilience, and resilience could help glaucoma patients improve their quality of life, even if they face adversity (15–18). The effect of resilience has been reported in many clinical conditions; however, little is known about its effect in glaucoma patients, especially in the Chinese population.

Social support refers to the care and support that people feel from others, including supervisor support and objective support. The literature indicates that social support could reduce the tension caused by stressful events (19–21), and it acts as a protective factor, which is necessary for the process of resilience to occur (22), indicating that social support might be closely related to resilience. Social support (especially subjective support) also has a significant correlation with quality of life (23, 24).

However, to the best of our knowledge, few studies have explored the resilience and social support of glaucoma patients in China. Furthermore, few studies have examined the relationships among resilience, social support, and quality of life in glaucoma patients. Therefore, this study aimed to explore the relationships among resilience, social support, and quality of life in glaucoma patients and test if resilience mediates the relationship between social support and quality of life.

## Materials and Methods

### Participants

A total of 120 patientsi with primary glaucoma receiving treatment at the Department of Ophthalmology (both outpatient and inpatient) at Xiangya Hospital affiliated with Central South University from May 2018 to November 2018 were enrolled. The inclusion criteria were as follows: aged 18 years or above; diagnosed with primary glaucoma more than 6 months ago; intraocular surgery or glaucoma medical treatment history of more than 3 months; more than 2 weeks after ocular laser surgery (with stable intraocular pressure and visual field damage during this period); normal cognitive state; volunteered to participate in the study. The exclusion criteria were as follows: combined systemic disease; suffering from mental illness or cognitive dysfunction; suffering from acute eye disease or other chronic eye diseases. The sociodemographic characteristics of the 120 patients with primary glaucoma are shown in Table 1.

**Table 1:** Sociodemographic characteristics of 120 patients with primary glaucoma.

**Variables**		
Age (years)	56.9	(22–80 years)
Male	57	(47.5%)
Glaucoma family history	17	(14.2%)
**EDUCATION LEVEL**		
Primary school or below (0–6 years)	36	(30%)
Secondary school (7–12 years)	43	(35.8%)
University or above (>13 years)	41	(34.2%)
**RESIDENCE**		
Urban	76	(63.3%)
Rural	44	(36.7%)
**MARITAL STATUS**		
Married	107	(89.2%)
Divorced or widowed	13	(10.8%)
**MONTHLY INCOME (RMB/MONTH)**		
<3,000	57	(47.5%)
3,000–5,000	38	(31.7%)
>5,000	25	(20.8%)
**WORKING CONDITION**		
Employed	65	(54.2%)
Unemployed or retired	55	(45.8%)

*Data are expressed as mean (range) or frequency (percent)*.

### Procedures

The investigation was approved by the Ethics Committee of Xiangya Hospital. When the investigation was officially carried out, the investigator introduced the purpose and method of the study to the patients who met the inclusion criteria and had signed the informed consent form. The questionnaires were issued to patients before the treatment or surgery, and the subjects were guided by the standard guidance to complete the questionnaire independently according to their actual situation and subjective feelings. For patients with special conditions who could not fill out the questionnaire by themselves, a qualified physician read the contents of the questionnaire for them word by word, the patient chose the answers, and then the researcher recorded the answers on the questionnaire.

### Survey Scales

1. Our self-compiled general condition questionnaire was used to collect patient information, including age, gender, marital status, education level, and illness duration.
2. The Glaucoma Quality of Life-15 (GQL-15) questionnaire was used to measure quality of life in glaucoma patients. The GQL-15 is a 15-item scale with 4 factors developed by Nelson et al. (25). The GQL-15 covers 4 major visual impairment problems: central vision and near vision (2 items), peripheral vision (6 items), glare and dark adaptability (6 items), and outdoor activity ability (1 item).Some researchers (26, 27) have evaluated the GQL-15 as the most clinically relevant and useful glaucoma patient-specific quality of life scale.
3. The Connor-Davidson Resilience Scale (CD-RISC) measures resilience using a resilience table designed by American psychologists Connor and Davidson (28). The scale consists of 3 factors (toughness and control, strength, and optimism), with a total of 25 items, all of which are scored on a scale from 1 to 4, for a maximum total score of 100. The higher the score, the better the individual's resilience. The scale has been widely used and validated in clinical practice (29).
4. The Perceived Social Support Scale (PSSS) was compiled by Zimet et al. and has 12 items covering 3 dimensions: family support, friend support, and other support. The items are scored on a scale from 1 to 7, and the total score is used to assess the overall amount of social support perceived by the individual (30).

### Statistical Analysis

SPSS 22.0 was used for statistical analysis. Measurement data were expressed as *M* ± *SD*. Correlation analysis was performed using the Pearson correlation test. Regression analysis was performed using stepwise regression analysis. The mediating effect analysis was tested using the bootstrap method by running the PROCESS plugin in the SPSS software. The threshold for significance was set at *p* < 0.05, corrected for multiple comparisons when needed.

## Results

Table 2 shows the descriptive statistics of the patients' resilience, social support, and quality of life scores, including the total score, near vision, peripheral vision, glare and dark adaptation, and outdoor activity ability as assessed by the GQL-15.

**Table 2:** The perceived social support, resilience, and quality of life scores of glaucoma patients.

**Items**	**Scores (*M* ± *SD*)**
Resilience	61.75, 9.35
Social support	53.19, 8.76
Total GQL-15 score	29.12, 13.27
Central and near vision	26.55, 26.81
Peripheral vision	17.92, 21.28
Glare and dark adaptability	28.45, 22.73
Outdoor mobility	14.89, 24.32

The results showed that there were positive correlations among the total score as well as each dimension's score on the GQL-15, the resilience score, and the social support score. There was a positive correlation between the total resilience score and the total PSSS score as well as the score on each dimension of the PSSS. All of the correlations were statistically significant (*p* < 0.01), as shown in Table 3. Regression analysis was performed with gender, age, marital status, education level, and illness duration used as control variables, resilience as the independent variable, and GQL-15 scores as dependent variables. The results of the regression analysis (Table 3) indicated that both resilience and social support could positively predict quality of life (β = 0.435 and β = 0.358, respectively), and the total explanatory quantity of the two variables was 38.9%.

**Table 3:** Correlation analysis of glaucoma patient quality of life, perceived social support, and resilience.

**Variables**	**Resilience**	**Social support**	**Total GQL-15 score**	**Central and near vision**	**Peripheral vision**	**Glare and dark adaptability**	**Outdoor mobility**
Resilience	1						
Social support	0.454^**^	1					
Total GQL-15 score	0.643^**^	0.418^**^	1				
Central and near vision	0.738^*^	0.531^*^	0.643^*^	1			
Peripheral vision	0.633^**^	0.547^*^	0.625^**^	0.713^*^	1		
Glare and dark adaptability	0.544^*^	0.572^*^	0.674^*^	0.589^**^	0.655^*^	1	
Outdoor mobility	0.411^*^	0.468^*^	0.453^*^	0.636^*^	0.724^*^	0.590^*^	1

* *p < 0.05*,

** *p < 0.01*.

**Figure 1:**
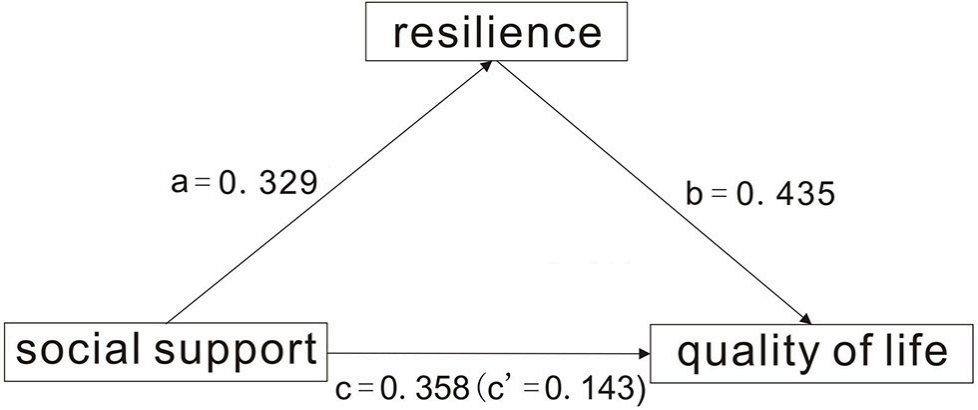
The model of mediation for the impact of social support toward quality of life in glaucoma with the mediation impact of resilience.

Next, resilience was set as a mediator, social support as the independent variable, and quality of life as the dependent variable. A bootstrap test was performed with 2,000 iterations of random sampling. The results showed that the mediating effect of resilience on the relationship between social support and quality of life was 0.143, and the 95%CI did not contain 0 [0.1031, 0.2432], indicating that the mediating effect was significant. After controlling the mediator resilience, the direct effect size of quality of life, which corresponded to the independent variable social support, was 0.435, and the 95%CI did not contain 0 [0.3321,0.5028] (Table 4). This result suggested a partial mediating effect of resilience on the relationship between social support and quality of life (Figure 1).

**Table 4:** The regression results of the effects of perceived social support and resilience on quality of life.

**Independent variable**	**Partial regression coefficient**				***R***^**2**^	***F***	***p***
	**β**	**SE**	***T***	***p***			
Constant	40.102	2.467	9.387	<0.01	0.389	138.542	<0.01
Resilience	0.435	0.045	8.765	<0.01			
Social support	0.358	0.031	8.411	<0.01			

## Discussion

Studies have shown that glaucoma is the second leading cause of blindness worldwide (31). Glaucoma causes a large financial burden on patients, and vision problems also affect all aspects of patients' lives. This study showed that patients with glaucoma have poor social support and low quality of life, consistent with previous studies (27). Few related studies on the resilience of glaucoma patients have been conducted at home and abroad. The results of our study showed that the level of resilience in glaucoma patients is lower than that in healthy people (32). Our findings indicate that there is an urgent need to improve the resilience, social support, and quality of life of glaucoma patients.

This study found positive correlations between the total scores as well as the scores on each dimension of the GQL-15, the resilience score, and the perceived social support score. Related studies (12, 33) also showed that individuals with high resilience would have positive adaptive behaviors in the face of traumatic events, which could promote individual subjective well-being and quality of life. A cognitive assessment of the availability and adequacy of support from family, friends, or others in this study showed that social support was positively correlated with quality of life in glaucoma patients, which was consistent with many previous studies (34, 35). As with other groups, the more social support glaucoma patients receive or perceive, the more they can experience subjective well-being; social support can prevent or alleviate stress responses, increase healthy behavior patterns, prevent the decrease of subjective well-being, and improve quality of life (36–38). The results also showed that the resilience and social support of glaucoma patients were positively correlated. Therefore, we can increase the use of social support by increasing resilience and improve patients' resilience by increasing social support, thereby improving the quality of life of glaucoma patients.

Multivariate stratification analysis showed that the level of resilience of glaucoma patients had a partial or complete mediating effect on the relationship between social support and quality of life. Resilience and social support could both predict the patients' quality of life. This suggested that resilience plays an important role in improving glaucoma patients' utilization of social support and quality of life. The quality of life of glaucoma patients could be further improved by increasing their resilience and social support. For example, Costa et al. (39) found that resilience could moderately improve the quality of life of patients with rectal cancer, and social support had a strong and direct comprehensive impact on patients' quality of life (including social, physical, and emotional quality). A study by Ong et al. (40) found that improving resilience and social support could significantly reduce the caregivers' burden. Therefore, increasing the resilience and social support of glaucoma patients is a novel and significant way to improve their quality of life.

In conclusion, the quality of life of glaucoma patients is generally poor and affected by hospital stays, sick time, and resilience. In view of the fact that resilience can affect the quality of life of patients to a large extent and can be intervened in, medical workers should carry out multilevel interventions for glaucoma patients from individual, family, social, and cultural perspectives to achieve the purpose of improving their quality of life.

There are some limitations to this study. The main limitation of this study is the lack of a control group. Furthermore, the number of subjects was small, and the outcomes included only remissions or partial remissions of glaucoma. Finally, the subjects needed to be capable of understanding the questionnaire. Therefore, the range of glaucoma patients covered in this study was narrow. Nonetheless, we believe that our findings can provide new ideas and research directions for improving the quality of life of glaucoma patients.

## Ethics Statement

All of the participants were aware of the purpose of the study. All of the research protocols were explained to the participants, and they signed a written consent form approved by the local IRB (Xiangya Hospital of Central South University of Hunan Province, Changsha, China) before the study.

## Author Contributions

YW, YZ, and XX conceived and designed the experiments. YW, YZ, SX, XW, and QC conducted the experiments and collected data. YW and YZ analyzed the results. YW wrote the main manuscript text. All of the authors reviewed the manuscript.

### Conflict of Interest Statement

The authors declare that the research was conducted in the absence of any commercial or financial relationships that could be construed as a potential conflict of interest.
